# Perception and management of fever in infants up to six months of age: A survey of US pediatricans

**DOI:** 10.1186/1471-2431-10-95

**Published:** 2010-12-22

**Authors:** Antoine C El Khoury, Emily Durden, Larry Ma, Leona E Markson, Andrew W Lee, Yinghui Duan, Kathleen Foley

**Affiliations:** 1 Merck & Co., Inc., West Point, PA, USA; 2 Covance, San Diego, CA, USA; 3 Thomson Reuters, Washington DC, USA; 4 Lehigh University, Bethlehem, PA, USA

## Abstract

**Background:**

A fever is an increase in the body's temperature above normal. This study examined how US pediatricians perceive and manage fever generally versus fever occurring after vaccination in infants up to six months of age.

**Methods:**

A web-based survey of 400 US pediatricians subscribing to the Physician Desk Reference was conducted in December 2008. Data were collected on the respondents' socio-demographics, number of years in practice, type of practice, their definition of fever severity in infants, and their recommendations for managing fever. Generalized Estimating Equations were used to estimate the odds of a pediatrician recommending an emergency room visit (ER) or a hospital admission, office visits, or other treatment option, as a function of infant's age, temperature, whether the infant has recently received a vaccine, and whether the fever was reported during or after office hours, adjusting for practice type and socio-demographic variables.

**Results:**

On average, the 400 responding pediatricians' (64% were female, average age of 49 years, years in practice = 20 years) threshold for extremely serious fever was ≥39.5°C and ≥ 40.0°C for infants 0-2 month and >2-6 month of age respectively. Infants were more likely to be referred to an ER or hospital admission if they were ≤ 2 months of age (Odds Ratio [OR], 29.13; 95% Confidence interval [95% CI], 23.69-35.82) or >2-4 months old (OR 3.37; 95% CI 2.99-3.81) versus > 4 to 6 months old or if they had a temperature ≥ 40.0°C (OR 21.06; 95% CI 17.20-25.79) versus a temperature of 38.0-38.5°C. Fever after vaccination (OR 0.29; 95% CI 0.25-0.33) or reported during office hours (OR 0.17; 95% CI 0.15-0.20) were less likely to result in referral to ER or hospital admission.

**Conclusion:**

Within this sample of US pediatricians, perception of the severity of fever in infants, as well as the response to infant fever are likely to depend on the infant's age. Recommendations for the management of fever in infants are likely to depend on fever severity level, the infant age, timing in relation to recent vaccination, and the time of day fever is reported. Our results indicate that US pediatricians are more concerned about general fever than fever following vaccination.

## Background

Fever is defined as an increase in the body's internal temperature to levels above the normal range. Fevers are usually the result of a viral or bacterial infection [1,2], but may also be caused by varied non-infectious conditions. In infants, fever warrants special attention as dehydration that may result from fever may compromise an infant's health more quickly than that of an adult [1,3], and may also be the sole indicator of certain life threatening conditions including meningitis and sepsis. Infants with fever are typically irritable and do not eat or sleep well. Because of the potentially serious causes of fever in infants, and the associated discomfort it elicits, fever is among the most common reasons parents seek medical attention for their infants [4].

Fever is commonly observed following vaccination. Vaccine-associated fevers usually begin within 24 hours of vaccine administration and last two to three days [5,6]. The American Academy of Pediatrics (AAP), the American Academy of Family Physicians (AAFP), and the Advisory Committee on Immunization Practice (ACIP) recommend administration of up to 9 different vaccine antigens during the first six months of life [7], many of which may cause fever following administration. However, it should be noted that high fever (greater than 39.5°C) occurs in less than 1% of infants [8].

US practice guidelines for the management and treatment of febrile infants have been met with controversy [9,10]. Differences of opinion among physicians regarding the recommended diagnostic response and the value of empiric antibiotic treatment have been documented, with hospital-based providers more often favoring reliance on testing and treatment than physicians in private office settings [9,10]. Such work evaluating physicians' perception and approach to the management of fever in infants is now quite dated, however [10-12]. Beliefs among some pediatricians have changed after the introduction of pneumococcal conjugate vaccines [13]. Because of the high efficacy of the pneumococcal vaccine against invasive infections of *Streptococcus pneumoniae*, some pediatricians have practiced less intensive diagnostic procedures for the standard work-up of fever [13].

This study provides current data on pediatricians' perception and management of fever in infants from birth to six months of age, including perceptions of severity according to body temperature and infant age. Preferred management or treatment strategies for different fever ranges, ages, and vaccination status, were surveyed. This study is designed to inform the readers on contemporary US pediatricians' perception and referral practices to ER and hospital admissions related to fever management in infants. It is not designed to provide guidance on treatment practices.

## Methods

A web-based survey was conducted in December 2008 to examine US pediatricians' perception and management of fever among infants up to 6 months of age. The survey instrument was developed to address the following specific research questions:

1- How do US pediatricians characterize the severity of fever, based on infant age?

2- How does the management of fever in infants vary, according to infant age and temperature range?

3- Does the management of fever in infants vary when the fever follows vaccination?

4- Does the management of fever vary by practice setting?

### Sampling and data collection

The subscriber list of the Physician's Desk Reference (PDR) served as the sampling universe for this study. (PDR, by Thomson Reuters at Montvale, NJ). This list consists of over 250,000 clinicians across the United States, including approximately 35,600 pediatricians. Pediatricians who had previously indicated interest in volunteering to participate in survey-based studies were identified as potential respondents to the study survey. A randomly computer generated sample of these candidates was initially selected and letters soliciting their participation in the survey were sent via electronic mail. Because this initial recruitment effort did not yield a satisfactory response within the predetermined timeframe, two additional randomly computer generated samples of potential study candidates were drawn from the universe of pediatricians from the PDR and additional letters were distributed via electronic mail.

After the potential respondents provided electronic consent to participate in the study, confirmation of their eligibility occurred through a series of screening questions. One of the requirements was that potential respondents should be providing primary pediatric care to infants 6 months of age or younger. Finally, eligible consenting participants received a hypertext link to the web-based, self-administered questionnaire via electronic mail. Data collection ceased once a sample of 400 respondents was obtained which occurred after three days following the initial recruitment effort. Respondents received an honorarium payment of 40 US dollars for their participation.

### Institutional Review Board

The New England Institutional Review Board (NEIRB) reviewed and approved this study. Data collection was not initiated until approval of the NEIRB was received.

### Survey Design

A single survey was developed to evaluate US pediatricians perception and management of fever in infants.

Respondents were referred to a hypothetical well-appearing infant with a fever of unknown source and asked to rate the severity of fever assuming that the temperature was taken by rectal measurement and identify temperatures to match the following categories: mild, moderate, serious and extremely serious fever for each of the following age groups: birth to 2 months of age, 2 to 4 months of age, and 4 to 6 months of age. Age categories were chosen based on the most common vaccination schedules for infants in the US. Respondents were also asked to select one of six recommendation for the management of fever in otherwise healthy febrile infants in four different scenarios: parent calls during office hours, parent calls after office hours, parent calls during office hours regarding a fever within 48 hours following a vaccination, and parent calls after office hours regarding a fever within 48 hours following a vaccination. The six choices for recommendations were: (1) Manage at home without antipyretic medication; (2) Manage at home with antipyretic medication; (3) Manage at home with antipyretic medication and an office visit within 3 days; (4) Manage at home with antipyretic medication and an office visit the same day/next day; (5) Emergency Room (ER) visit immediately; and (6) Hospital admission immediately. The survey also collected data on respondents specialty, type of practice (solo, group office or Health Management Organization setting), number of years in practice, socio-demographic information, whether pediatricians rely on practice guidelines for the management of fever, the provision of education to parents regarding the management of fever, and geographic location of practice.

The survey was programmed into a web-based application hosted with redundant fail-over web servers and databases to ensure security of the data. Data validation logic was pre-programmed into the survey so that illogical and out-of-range data could not be entered. Initial web-based versions of the survey were reviewed, validated and tested by three physicians.

### Statistical Analysis

All variables and data were summarized using descriptive statistics. Multivariate logistic regression was conducted using Generalized estimating Equations (GEE) to estimate the odds of recommending a hospital admission or ER visit as a function of infant age, temperature, whether the infant recently received a vaccine, and whether the fever was reported during or after office hours, adjusting for practice type and socio-demographic variables.

The GEE model was used because it adjusts for the correlation due to the multiple responses from the same pediatrician. The recommendations provided by pediatricians were grouped into two categories: 1) hospital admission or ER visit; 2) all other recommendations (manage at home without antipyretic medication, manage at home with antipyretic medication, manage at home with antipyretic medication and an office visit within 3 days, and manage at home with antipyretic medication and an office visit the next day). Odds ratios (ORs) were provided along with the 95% confidence intervals (95% CI). Statistical significance was assessed at a two-sided 0.05 significance level. SAS version 9.13 [SAS Institute, Cary, NC] was used for data analysis.

## Results

In total, 17,392 pediatricians were invited to participate in the survey and the final sample comprised of 401 respondents (the target sample was 400 however there was one additional response that was reported).

### Respondent Characteristics

Demographic and practice characteristics of respondents are presented in Table 1. About two thirds of the respondents (64.3%) were female, the median age was 50 years and the mean age was 49.6 ± 10.6 years. Nearly three quarters of the respondents (73.8%) practiced in private group setting, 13.5% practiced in private solo setting, and 12.7% practiced in a Health Management Organization (HMO) setting. Additionally, most respondents practiced in a community-based setting (82.8%) rather than in a teaching/research institution (17.2%). Slightly more than half of the respondents (52.1%) practiced in suburban area, while 40.4% reported practicing in an urban area and 7.2% in a rural area. The largest proportion of respondents resided in the Western US (37.2%), followed by the South (30.4%), Northeast (20.4%), and Midwest (11.7%). The average number of years practicing pediatric medicine in the sample was 19.7 ± 10.4 years.

**Table 1:** Demographic Characteristics of Pediatrician Respondents

Demographic Characteristics	N	%
**Number of Respondents**	401	

**Gender (%)**		
Female	258	64.3%
Male	143	35.7%

**Type of Practice (%)**		
Private solo office	54	13.5%
Private group practice	296	73.8%
HMO setting/salaried through a health plan	51	12.7%
Missing	0	0.0%

**Practice Setting (%)**		
Community-based	332	82.8%
Teaching/research institution	69	17.2%
Missing	0	0.0%

**Geographic Region (%)**		
Within a city/in an urban area	162	40.4%
Surburban	209	52.1%
Rural	29	7.2%
Missing	1	0.2%

**Census Region (%)**		
Northeast	82	20.4%
South	122	30.4%
West	149	37.2%
Midwest	47	11.7%
Missing	1	0.2%

### Respondents' Perception of Seriousness of Fever

**Figure 1:**
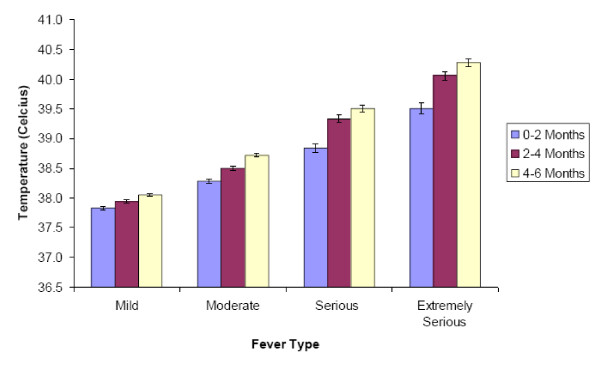
**Temperatures at Which Pediatricians Consider Infants to have Mild, Moderate, Serious and Extremely Serious Fever, by Infant Age**. • Bars refer to 95% CI

Pediatricians' categorization of fever is shown in Figure 1. On average, across the three age groups (0-2 months, 2-4 months, and 4-6 months), respondents indicated that they considered temperatures between 37.8-38.1°C to be mild, those between 38.3-38.7°C to be moderate, those between 38.8-39.5°C to be serious, and those between 39.5-40.3°C to be extremely serious. The temperature at which fevers were considered to be more serious increased with infant age. For example, a fever of 39.5 ± 0.9°C (mean ± S.D) was considered to be extremely serious for infants 0-2 months of age, while a fever of 40.3 ± 0.6°C (mean ± S.D) was considered extremely serious for infants 4-6 months of age. For a given category of fever, respondents consistently indentified a lower mean temperature for the younger age ranges, but the differences were not statistically significant for any of the categories.

### Predictors of Recommendation for Fever Management

Table 2 shows results of the multivariate GEE analysis. Results indicated that infant age, body temperature as well as vaccination status were all significant predictors of the respondents' recommendations for fever management. Participating pediatricians were more likely to recommend hospital admission or ER visit to parents if febrile infants were younger: 0-2 months of age (adjusted OR 29.13; 95% CI 23.69-35.82), or 2-4 months of age (adjusted OR 3.37; 95% CI 2.98-3.81) compared with infants 4-6 months of age. The likelihood of recommending hospital admission or ER visit increased with infant body temperature (Table 2). Respondents were less likely to recommend hospital admission or ER visit to parents with febrile infants who called regarding a fever following vaccination (adjusted OR 0.29; 95% CI 0.25-0.33) compared with no recent vaccination. Pediatricians were less likely to recommend a hospital admission or ER visit when parents called during office hours (adjusted OR 0.17; 95% CI 0.15-0.20) compared with calling after office hours.

**Table 2:** Odds of a Pediatrician Recommending a Hospital Admission or ER Visit

Characteristic	OR (95% CI)
**Infant Characteristics**	

**Age, month**	
0-2	29.13 (23.69-35.82)**
2-4	3.37 (2.98-3.81)**
4-6	1.00

**Teperature,°C**	
> = 40	21.06 (17.20-25.79)**
39.50-39.99	7.87 (6.71-9.22)**
39.0-39.49	3.68 (3.27-4.16)**
38.50-38.99	1.65 (1.53-1.79)**
38.0-38.49	1.00

**Vaccination Status**	
Vaccinated	0.29 (0.25-0.33)**
Not Vaccinated	1.00

**Time of Parent's Call**	
During Office Hour	0.17 (0.15-0.20)**
After Office Hour	1.00

**Pediatrician Characteristic**	
**Gender**	
Female	1.37 (1.02-1.84)*
Male	1.00

**Practice Characteristics**	
**Type of Practice**	
Solo	0.61 (0.35-1.07)
Group Office	0.47 (0.36-0.69)**
HMO setting	1.00

**Practice Setting**	
Community Based	0.98 (0.67-1.43)
Teaching/Research Institution	1.00

**Geographic Region**	
Urban	4.17 (1.22-14.35)*
Suburban	3.62 (1.18-11.08)*
Rural	1.00

**Census Region**	
Midwest	1.88 (1.10-3.23)*
Northeast	1.62 (1.02-2.58)*
South	1.43 (0.96-2.11)
West	1.00

CI, confident interval; OR, odds ratio.

*: p < 0.05

**: p < 0.001

Female respondents (adjusted OR 1.37; 95% CI 1.02-1.84), those practicing in urban (adjusted OR 4.17; 95% CI 1.22-14.35) and suburban areas (adjusted OR 3.62; 95% CI 1.18-11.08), and those practicing in the Midwest (adjusted OR: 1.88; 95% CI 1.10-3.23) and Northeast (adjusted OR 1.62; 95% CI 1.02-2.58) were more likely to recommend hospital admission or ER visit compared with male respondents, those practicing in rural areas, and those practicing in the West, respectively. Respondents practicing in a group office setting were less likely to recommend hospital admission or ER visit compared with those in HMO setting (adjusted OR 0.47; 95% CI 0.36-0.69). Respondents' age, number of years in practice, and the percentage of respondent's patients enrolled in private insurance were not significant predictors of the respondents' recommendations.

### Recommendation for Fever Management Following Vaccination

Nearly three-quarter (72.3%) of the sample reported that their recommendations for fever management would differ if fever followed vaccination rather than if fever was of general nature. However, when asked at what temperature antipyretic medication would be recommended for general fever and fever after vaccination, participating pediatricians responded without significant difference between the two clinical scenarios. In general, respondents indicated that they would recommend the use of antipyretic medications at temperatures of about 38.3°C, regardless of whether fever followed vaccination or not. The average temperatures at which respondents indicated they would recommend antipyretic medications for both general fever and those following vaccination increased very slightly with infant age. The recommendation to manage fever within the 48 hours following vaccination at home without antipyretic medication was not a common choice for most respondents. Over 97.3% reported never recommending this for infants with extremely serious fever and 56.1% reported never recommending this option for infants with mild fever. Managing at home with antipyretic medication was more likely to be recommended for infants with mild or moderate fever.

### Reliance on Practicing Guideline and Parents' Education About Fever Management

Most respondents (89.5%) reported some reliance on the fever management guidelines set forth by the American Academy of Pediatrics (AAP) in their response to fever in infants; only 10.5% reported that they did not use the guidelines of the AAP in their practice.

Most respondents (85.3%) reported that they "always" or "usually" explained to parents how to manage infant fever during well-baby visits; and even higher proportion (95.8%) reported that they "always" or "usually" explained to parents how to manage infant fever during a vaccination visit.

## Discussion

The objective of this study was to examine U.S. pediatricians' perceptions and management of fever in infants from birth to six months of age. Pediatricians providing primary care were recruited to participate in the study. Participating pediatricians were asked to complete a survey that contained questions regarding the management and perception of fever in infants. Special emphasis was placed on fever following vaccination.

In general, respondents seem to be less concerned when fever follows a vaccination compared to fever of general nature and expressed consensus regarding the temperatures they considered to represent mild to extremely serious fevers for the different infant age groups.

On average pediatricians in our sample were less likely to recommend ER visit or hospital admission to parents of infants with fever occurring within 48 hours after immunization versus those with fever not after immunization. Data collected from routine childhood vaccines have shown that fever typically occurs in 1%-10% of infants after vaccination[14], but the rate can increase as high as 30% to about 50% among those who received multiple vaccines [14,15]. As a benign symptom, fever following immunization generally resolves without long-term sequelae [16]. This may explain why pediatrician respondents were less concerned about fever after immunization.

In general, the intensity of treatment or management recommendations provided by respondents increased with higher levels of fever and is inversely related to infant age. Whittler et al.[11] conducted a survey in 1998 to examine pediatricians' recommendation for infants with a body temperature of 40.3°C. In their study, 96% of the responders recommended hospital admission for the 3 week old febrile infants and 62% recommended hospital admission for 7 week old febrile infants. In our study, 86.0% of respondents recommended immediate ER visit or hospital admission for infants 0-2 months of age with a body temperature greater than 40.0°C. Whittler et al. found that 6% of respondents recommended hospital admission for 4-month-old infants with a fever of 40.3°C, while 31.1% of the respondents in our study recommended hospital admission for the same scenario. The difference in these results may be due to the different designs of the questionnaires for the two surveys. In our survey, all recommendations were based on a scenario of parents' calls, while in Whittler's study the participating pediatricians were provided a scenario of clinic visit. We found that physician respondents were more inclined to recommend an ER visit in response to infant fever after office hours rather than during office hours. Results indicate that a significant portion of recommendations for ER visits would have been recommendations for a same day office visit had the fever occurred during office hours. These results may have practical implications for the

organization of the emergency departments during off-office hours and may suggest that specific educational programs for the management of fever in infants are needed in the US.

Other studies in the US as well as outside the US reported variable compliance rates to current guidelines for managing fever. Young conducted a case scenario survey for primary care physicians in Utah [17]. The results of the study showed that compliance with guidelines vary from 9.6% to 75% depending on the age of the febrile child. Gabriel et al found that pediatricians and emergency department physicians would order fewer tests and would prescribe less antibiotics to febrile children who have been vaccinated previously with pneumoccoal conjugate vaccine [18]. Chiappini et al found that the management of well-appearing febrile infants varies across pediatricians in Italy after the introduction of the pneumococcal conjugate vaccine [13]. Although most pediatricians in our study reported some reliance on the fever management guidelines set forth by the American Academy of Pediatrics (AAP), it is apparent that the general attitude of pediatricians towards fever management has been changing since the introduction of the pneumococcal conjugate vaccine.

Our findings suggest that pediatricians recommend antipyretic medications at temperatures of about 38.3°C, regardless of whether fever followed vaccination or not. Prescription of antipyretics at a certain body temperature level is not specifically or routinely recommended by the AAP, NICE, WHO or the Italian guidelines for the management of fever in children [19-21]. Generally, the use of antipyretics in children is recommended only when the fever is associated with evident discomfort (eg, prolonged crying, irritability, reduced activity, reduced appetite, disturbed sleep) and not for a given temperature level. It is important to note that neither the level of fever, nor the response to antipyretics, is a predictive factor for the cause of fever.

There are some limitations to consider when interpreting the results of this study. First, the questionnaire used to collect data on the pediatrician perception and recommendation for fever management did not include aspects related to the infant behavior. Pediatricians may want to know more about the symptoms and the behavior of the infant in order to give a recommendation for managing fever. Second, as with any survey based on a convenience sample, the results obtained from this sample may not be a representative sample of all the pediatricians in the US. The sampling design of this survey was a "first-come, first served" to a web link. This may have introduced bias in the final sample since fast responders and/or those with more readily available web access were more likely to be included. This design precludes measurement of the true response rate. Third, although we hoped to have a balanced representation from all the US regions, we still had an underrepresentation from the Midwest. This may introduce some bias if there are differences in managing fever across regions.

Finally, this study focused on the definition of fever severity based on its level and infant's age. Although in special circumstances high fever may be a predictive factor for severe bacterial infection, especially in children under three months of age, in itself, the degree of fever should not be taken as an indicator of the risk for severe disease [21].

## Conclusion

Our results indicate that US pediatricians are more concerned about general fever than fever following vaccination. Management and definitions of fever severity by pediatricians in our sample depend on infant age. Recommendations for managing fever were dependent on the fever level, infant age, timing in relation to vaccination, and the time of day fever was reported. These results may be valuable when developing fever management guidelines and when estimating the burden of managing fever in the US.

## Competing interests

Financial support for this manuscript was provided by Merck & Co., Inc. AEK, LM, LEM and AWL are employees of Merck & Co., Inc. Thomson Reuters received funds from Merck & Co., Inc. to conduct the study. At the time the study was conducted, ED was an employee of Thomson Reuters and YD was completing a fellowship at Lehigh University which was funded by Merck & Co., Inc.. KF is an employee of Thomson Reuters.

## Authors' contributions

AEK conceived the study and participated in its design, coordination and analysis of the data and took the lead on drafting the manuscript. ED and KF led the administration of the survey, and participated in the design and coordination of the study and analysis of the data. LM participated in the design of the study, conducted the statistical analysis and helped in drafting the manuscript. AWL participated in the conception and design of the study and drafting of the manuscript. YD helped in drafting the manuscript. LEM helped in the design of the study and drafting the manuscript. All authors read and approved the final version of the manuscript.

## Pre-publication history

The pre-publication history for this paper can be accessed here:

http://www.biomedcentral.com/1471-2431/10/95/prepub
